# Supplementary material for: DIPPER, a spatiotemporal proteomics atlas of human intervertebral discs for exploring ageing and degeneration dynamics
Source: eLife. 2020 Dec 31;9:e64940. doi: 10.7554/eLife.64940 (PMC7857729; doi:10.7554/eLife.64940)
Supplement: Transparent reporting form [file elife-64940-transrepform.pdf]

eLIFE
1st Floor
24 Hills Road
Cambridge CB2 1JP, UK
P 01223 855340
W elifesciences.org
T @elife

eLife's transparent reporting form

We encourage authors to provide detailed information *within their submission* to facilitate the interpretation and replication of experiments. Authors can upload supporting documentation to indicate the use of appropriate reporting guidelines for health-related research (see EQUATOR Network), life science research (see the BioSharing Information Resource), or the ARRIVE guidelines for reporting work involving animal research. Where applicable, authors should refer to any relevant reporting standards documents in this form.

If you have any questions, please consult our Journal Policies and/or contact us: editorial@elifesciences.org.

Sample-size estimation

- You should state whether an appropriate sample size was computed when the study was being designed
- You should state the statistical method of sample size computation and any required assumptions
- If no explicit power analysis was used, you should describe how you decided what sample (replicate) size (number) to use

Please outline where this information can be found within the submission (e.g., sections or figure legends), or explain why this information doesn't apply to your submission:

We are providing genome-wide proteomic measurements of the human intervertebral discs from cadaveric samples and clinical samples. The cadaveric samples, which enables the ultra-high spatial resolution label-free proteomic measurements within and at multiple intact lumbar disc levels, consist of one young and one aged cadavers. In terms of inference of population-wide characteristics, they may not be representative. But they are considered to be of important point-reference values, given the rarity of such intact discs in the two age groups and how we designed along the lateral and anteroposterior axes (Figure 1D).

The clinical samples consist of multiple individuals (2 to 4) per age groups, for all three types of data (SILAC, degradome, and transcriptome).

The power issue matters primarily in our detection of differentially expressed proteins (DEPs). To tackle this, we required that the numbers of samples per group are 3 or more, in any comparison. Also, we converted the raw p-values to FDR (false discovery rate) by the Benjamini & Hochberg method, so that only FDR<0.05 were considered significant. This information can be found in the methods section.

Replicates

- You should report how often each experiment was performed
- You should include a definition of biological versus technical replication
- The data obtained should be provided and sufficient information should be provided to indicate the number of independent biological and/or technical replicates
- If you encountered any outliers, you should describe how these were handled
- Criteria for exclusion/inclusion of data should be clearly stated
- High-throughput sequence data should be uploaded before submission, with a private link for reviewers provided (these are available from both GEO and ArrayExpress)

eLife Sciences Publications, Ltd is a limited liability non-profit non-stock corporation incorporated in the State of Delaware, USA, with company number 5030732, and is registered in the UK with company number FC030576 and branch number BR015634 at the address 1st Floor, 24 Hills Road, Cambridge CB2 1JP | August 2014

Please outline where this information can be found within the submission (e.g., sections or figure legends), or explain why this information doesn't apply to your submission:

The datasets used in this paper include 94 proteomic (both static and dynamic) and transcriptomic profiling from 17 individuals. This was outlined in Figure 1A and Table 1, and detailed in Methods.

Specifically, the static proteome dataset comprises 66 proteome-wide profiles from 2 cadaveric samples; the dynamic proteome dataset comprises 14 profiles from 11 individuals (5 for SILAC and 6 for degradome); and the transcriptomic dataset comprises 8 genome-wide profiles from 4 individuals. More details are provided below.

Static spatial proteome samples (cadaver specimens; by LC-MS/MS)

- Number of individuals: n=2 (1 young and 1 aged; neither of whom is known to have scoliosis nor degeneration diagnosis.).
- Number of disc levels per individual: 3 levels (L3/4, L4/5, and L5/S1).
- Number of separate regions per disc: 11.
- Total number of samples per individual: 3 × 11 = 33.
  (Note that these 33 samples are not considered technical replicates, as they measured proteomes of separate structures.)
- Total number of proteome-wide measurements for all cadavers: n=33 × 2 = 66.
- In performing LC-MS/MS, each of these 66 samples were fractionated into 4 fractions and measured on mass spectrometer. The data coming from these 4 fractions were pooled to form a single proteomic profile for the respective sample.
- These 66 samples measure different levels, regions (NP, NP/IAF, IAF, and OAF), and directions of the discs in two individuals, as outlined in Figure 1C-D; and summarised below:

Cadaver samples	Number NP regions	Number NP/IAF regions	Number IAF regions	Number OAF regions	(Row-sum)
Young (16, M)	3	12	6	12	(33)
Aged (59, M)	3	12	6	12	(33)
(Column-sum)	(6)	(24)	(12)	(24)	(66)

- The raw data and metadata can be found in the PRIDE database under PXD017740 (A proteomic architectural landscape of the healthy and aging human IVD, username: reviewer85869@ebi.ac.uk, password: yPQEf7bP). No raw data was excluded.

Transcriptome samples (clinical samples; by microarray)

- Number of individuals: n=4 (2 aged, 2 young).
- Number of structures per individual: 2 (NP and AF).
  (*Note that the two data profiles from these two structures in each individual were again not considered technical replicates, because they measured samples from different structures and the effect of this structure information was to be analysed.*)
- Total number of genome-wide profiles: 4 × 2 = 8.

These samples were used to validate findings in the static proteomic data. The raw data and metadata can be found in NCBI GEO with accession number GSE147383.

SILAC samples (clinical samples; for measuring protein synthesis)

- Number of individuals: n=5 (4 young, 1 aged).
- Number of regions per individual: 1 or 2 (NP and/or AF).
- Total number of SILAC profiles: 8.

Each of these 8 samples has between 3 to 7 technical replicates, as summarised below.

Sample	Region	Technical replicates
YND148	AF	7
YND149	AF	3
YND151	NP	5

eLife Sciences Publications, Ltd is a limited liability non-profit non-stock corporation incorporated in the State of Delaware, USA, with company number 5030732, and is registered in the UK with company number FC030576 and branch number BR015634 at the address 1st Floor, 24 Hills Road, Cambridge CB2 1JP | August 2014

YND151	AF	4
YND152	NP	4
YND152	AF	4
AGD80	NP	4
AGD80	AF	4

For each of these samples, its technical replicates were pooled to form a SILAC profile.

The SILAC raw data and metadata can be found in the PRIDE database under PXD018193 (Actively synthesised proteins in human IVD, username: reviewer74353@ebi.ac.uk, password: ycTEODdw). No raw data was excluded.

Degradome samples (clinical samples; for measuring protein cleavage or degradation)

- Number of individuals: n=6 (2 young and non-degenerated, 4 aged or degenerated);
- Number of regions per individual: 2 (NP and AF).
- Total number of genome-wide degradome measurements: 6 × 2 = 12.

Note:

- Sample information is summarised in Table 1.
- The NP or AF samples were pooled for analysis, respectively.
  (ie: all young and aged NP in one experiment; and all young and aged AF in another experiment).
- Each experiment was run in technical duplicates on the mass spectrometer.

The raw data and metadata can be found in the PRIDE database under PXD018298 (The degradome of the human IVD, username: reviewer81572@ebi.ac.uk, password: UUN5J57Q). No raw data was excluded.

Statistical reporting

- Statistical analysis methods should be described and justified
- Raw data should be presented in figures whenever informative to do so (typically when N per group is less than 10)
- For each experiment, you should identify the statistical tests used, exact values of N, definitions of center, methods of multiple test correction, and dispersion and precision measures (e.g., mean, median, SD, SEM, confidence intervals; and, for the major substantive results, a measure of effect size (e.g., Pearson's r, Cohen's d)
- Report exact p-values wherever possible alongside the summary statistics and 95% confidence intervals. These should be reported for all key questions and not only when the p-value is less than 0.05.

Please outline where this information can be found within the submission (e.g., sections or figure legends), or explain why this information doesn't apply to your submission:

eLife Sciences Publications, Ltd is a limited liability non-profit non-stock corporation incorporated in the State of Delaware, USA, with company number 5030732, and is registered in the UK with company number FC030576 and branch number BR015634 at the address 1st Floor, 24 Hills Road, Cambridge CB2 1JP | August 2014

- In comparing protein numbers detected and their levels between young and aged static proteomes, we used the Wilcoxon test (Supplementary Figure S2 and S4), a nonparametric method that are known to be less sensitive to spurious observations.
- For detection of DEPs (differentially expressed proteins), we used two-tailed Student's *t* test for each protein, with the resulting p-values to be corrected for multiple testing by the false discovery rate (FDR), with a cut-off of FDR<0.05 to be considered significant. The DEPs were visualised in volcano plots in Figures 3-5 and Supplemental Figures S6,9-10; and listed in Supplemental Tables S2-4.
- The Pearson Correlation Coefficients (PCCs) were used where collinearities or correlations were reported, unless otherwise specified (e.g. Supplemental Figure S13E, where the Spearman correlation coefficient, or SCC, was also reported). P-values of PCCs or SPCs were obtained from the cor.test function of the R software.
- We have shown the median values on top of each violin in the violin plots of our Figures and supplemental figures, wherein the inter-quartile range were also visualised by the box and whisker plots.

We visualised raw data —protein intensities in log_2 (LFQ)—on selected sets of proteins (e.g. Figure 5H and Supplemental Figure S7) by heatmaps.

(For large datasets, or papers with a very large number of statistical tests, you may upload a single table file with tests, Ns, etc., with reference to sections in the manuscript.)

Group allocation

- Indicate how samples were allocated into experimental groups (in the case of clinical studies, please specify allocation to treatment method); if randomization was used, please also state if restricted randomization was applied
- Indicate if masking was used during group allocation, data collection and/or data analysis

Please outline where this information can be found within the submission (e.g., sections or figure legends), or explain why this information doesn't apply to your submission:

Group allocation does not apply to our submission because this is not a clinical study, or one that requires group allocation

Additional data files ("source data")

- We encourage you to upload relevant additional data files, such as numerical data that are represented as a graph in a figure, or as a summary table
- Where provided, these should be in the most useful format, and they can be uploaded as "Source data" files linked to a main figure or table
- Include model definition files including the full list of parameters used
- Include code used for data analysis (e.g., R, MatLab)
- Avoid stating that data files are "available upon request"

Please indicate the figures or tables for which source data files have been provided:

The custom scripts for processing and analysing the data have been made publicly available and are housed at http://github.com/hkudclab/DIPPER

An explorable web interface is developed and hosted at: http://www.sbms.hku.hk/dclab/DIPPER
